# A patient with metformin-associated lactic acidosis successfully treated with continuous renal replacement therapy: a case report

**DOI:** 10.1186/s13256-019-2311-5

**Published:** 2019-12-17

**Authors:** Hiroki Kinoshita, Machi Yanai, Koichi Ariyoshi, Motozumi Ando, Ryo Tamura

**Affiliations:** 1 Department of Emergency Medicine, Kobe City Medical Center General Hospital, 2-1-1, Minatojima-Minamimachi, Chuo-ku, Kobe, Hyogo 650-0047 Japan; 2 Division of Clinical Pharmacy, Faculty of Pharmaceutical Sciences, Kobe Gakuin University, Kobe, Japan; 3 Department of Pharmacy, Kobe City Medical Center General Hospital, Kobe, Japan

**Keywords:** MALA, Metformin, Lactic acidosis, CRRT, Continuous renal replacement therapy

## Abstract

**Background:**

Metformin has been widely used as a first-line agent to treat type 2 diabetes mellitus. Lactic acidosis is a rare but serious adverse effect in patients treated with metformin. Recent studies noted a correlation between metformin accumulation and lactic acidosis. Continuous renal replacement therapy for the treatment of metformin-associated lactic acidosis has been documented in some case reports; however, there is currently no specific treatment for metformin-associated lactic acidosis.

**Case presentation:**

A 70-year-old Japanese woman with type 2 diabetes mellitus presented to an emergency room with metformin-associated lactic acidosis. She was found to be hypotensive and laboratory examinations revealed severe lactic acidosis: pH 6.618, partial pressure of carbon dioxide in arterial blood 17.3 mmHg, bicarbonate 1.7 mmol/L, and lactate 18 mmol/L. Severe acidemia persisted despite supportive care including intravenously administered fluids, sodium bicarbonate, antibiotics, and vasopressors. Continuous renal replacement therapy was initiated in our intensive care unit. After dialysis for 3 days, her lactate level and pH value completely normalized. The concentration of metformin detected was 77.5 mg/L, which is one of the highest in metformin-associated lactic acidosis successfully treated without overdose.

**Conclusions:**

The present case had one of the highest metformin concentrations in metformin-associated lactic acidosis successfully treated with continuous renal replacement therapy, and serum metformin concentrations may be useful for the diagnosis of metformin-associated lactic acidosis. Metformin-associated lactic acidosis is a rare but important etiology of lactic acidosis. Continuous renal replacement therapy is advantageous for the treatment of hemodynamically unstable patients with metformin-associated lactic acidosis.

## Background

Metformin, a biguanide antihyperglycemic drug, has been widely used as a first-line agent to treat type 2 diabetes mellitus. It inhibits pyruvate carboxylase, which impairs the conversion of lactate to pyruvate, leading to impaired cellular respiration; this results in both lactate production and its impaired metabolism. Lactic acidosis is a rare but serious adverse effect in metformin-treated patients [1]. The incidence of metformin-associated lactic acidosis (MALA) is less than 1–9 cases/100,000 patient years [2]. The mortality rate is 30–50% [3]. Intermittent renal replacement therapy has been suggested as a treatment method for drug removal and the correction of severe acidemia; however, a standard treatment strategy has not yet been established [4]. Continuous renal replacement therapy (CRRT) and sustained low-efficacy dialysis (SLED) for the treatment of MALA have been documented in some case reports [5, 6]. The advantages of CRRT and SLED are improved tolerability by hemodynamically unstable patients. We describe a case of MALA that was successfully treated with CRRT.

## Case presentation

A 70-year-old Japanese woman with a history of type 2 diabetes mellitus presented to our emergency room with diarrhea, nausea, and vomiting for 3 days. She also had a history of colon polyps, depression, and chronic kidney disease (CKD) stage G3a. She had been taking metformin 1000 mg/day for more than 3 years without changing the dosage. Her condition was normal until 3 days before her admission when she started to have repeated diarrhea and vomiting. Her consciousness was deteriorating on the day of her admission. Her vital signs on arrival were as follows: blood pressure 71/56 mmHg, heart rate 85 beats per minute, temperature 35.7 °C, oxygen saturation 86% on room air, and respiratory rate 24 breaths per minute with Glasgow Coma Scale E4V4M5. Her initial laboratory examination revealed severe kidney injury with blood urea nitrogen (BUN) of 67.5 mg/dL and creatinine of 10.17 mg/dL and she had metabolic acidosis with a high lactate level: pH 6.618, partial pressure of carbon dioxide in arterial blood (PaCO_2_) 17.3 mmHg, bicarbonate (HCO_3_^−^) 1.7 mmol/L, and lactate 18 mmol/L (Table 1). Whole-body computed tomography showed maxillary sinusitis and a uterine fibroid, but no evidence of infection.

**Table 1:** Laboratory results on admission

Biochemistry		Complete blood count		Blood gas	
TP	6.2 g/dL	WBC	17,800/μL	pH	6.618
Alb	3.5 g/dL	Hb	9.7 g/dL	PaCO_2_	17.3 mmHg
BUN	67.5 mg/dL	Ht	35.7%	PaO_2_	71.1 mmHg
Cre	10.17 mg/dL	Plt	27.6 × 10^4^/μL	HCO_3_^−^	1.7 mmol/L
Na	143 mEq/L			AG	31.3 mmol/L
K	5.4 mEq/L			Lactate	18 mmol/L
Cl	100 mEq/L				
Ca	7.7 mg/dL				
Glu	99 mg/dL	Coagulation test			
T-Bill	0.2 mg/dL	PT-INR	1.13		
AST	79 U/L	PT%	80.5%		
ALT	52 U/L	APTT	13.9 seconds		
LDH	305 U/L				
CRP	0.31 mg/dL				

*AG* anion gap, *Alb* albumin, *ALT* alanine aminotransferase, *APTT* activated partial thromboplastin time, *AST* aspartate aminotransferase, *BUN* blood urea nitrogen, *Cre* creatinine, *CRP* C-reactive protein, *Glu* glucose, *Hb* hemoglobin, *HCO*_*3*_^*−*^ bicarbonate, *Ht* hematocrit, *LDH* lactate dehydrogenase, *PaCO*_*2*_ partial pressure of carbon dioxide in arterial blood, *PaO*_*2*_ partial pressure of oxygen in arterial blood, *Plt* platelets, *PT* prothrombin time, *PT-INR* prothrombin time-international normalized ratio, *T-Bill* total bilirubin, *TP* total protein, *WBC* white blood cells

**Fig. 1:**
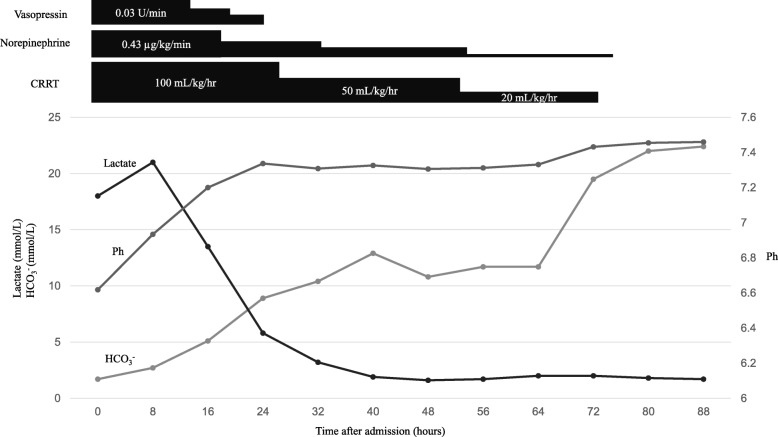
Clinical course of the patient. *CRRT* continuous renal replacement therapy, *HCO*_*3*_^*−*^ bicarbonate

Although supportive measures, including intravenously administered fluids, sodium bicarbonate, vasopressors, and antibiotics, were initiated, severe acidemia and hemodynamic instability persisted. Tracheal intubation was performed and she was transferred to the intensive care unit where she received urgent CRRT (continuous hemodiafiltration with a dialysate rate of 5000 mL/hour). Her lactate level and pH value completely normalized within 72 hours and, thus, CRRT and vasopressors were stopped (Fig. 1). She was extubated on day 6 and antibiotics were discontinued based on negative blood cultures on day 7. She left the intensive care unit on day 12 and was discharged on day 20. Her serum creatinine level before discharge was 1.11 mg/dL.

After discharge, she was treated as an out-patient with orally administered anti-diabetic drugs other than metformin and her serum creatinine level remained stable. Her metformin level before CRRT was 77.5 mg/L (therapeutic range 0.5–2 mg/L). Metformin concentrations were measured by high-performance liquid chromatography-tandem mass spectrometry using an Agilent 1260 Infinity HPLC system (Agilent Technologies, Santa Clara, CA, USA) with an InertSustainSwift™ C18 column (2.1 × 50 mm, 2.0 μm; GL Sciences Inc., Tokyo, Japan) and QTRAP® 4500 mass spectrometer (AB Sciex, Framingham, MA, USA) using isocratic elution of 10 mM ammonium acetate and acetonitrile at a ratio of 80:20 (v/v). The linearity of the calibration curve was between 6.25 and 100 mg/L with a determination coefficient of 0.999.

## Discussion and conclusions

We identified two important clinical issues. The present case had one of the highest metformin concentrations in MALA successfully treated with CRRT and serum metformin concentrations may be useful for the diagnosis of this condition. CRRT is an effective treatment for patients with MALA with hemodynamic instability.

Metformin blood concentrations ranging between 0.5 and 2.0 mg/L are within the therapeutic range, whereas those higher than 4.0 mg/L are generally considered to be toxic [1]. The causal link between metformin accumulation and lactic acidosis has been questioned [7, 8]. Previous studies reported a poor correlation, which may have reflected the suboptimal timing of sample collection [8]. Recent studies noted a correlation between metformin accumulation and lactic acidosis [9]. In the present case, the serum metformin level before CRRT was 77.5 mg/L. This level exceeded the lethal range of metformin concentrations (> 50 mg/L) [6, 10]. Moreover, this patient had most of the poor prognostic indicators in MALA, such as advanced age, arterial pH less than 7.35, the need for mechanical ventilation, and vasopressors [11]. Although a poor prognosis was expected, we successfully treated one of the highest metformin concentrations in MALA with CRRT. Our suspicion of MALA was supported by the high level of metformin. Although plasma metformin concentrations are not easily available in all laboratories, it is important to ensure specimen storage because plasma metformin concentrations measured in the emergency room will contribute to an accurate diagnosis [12].

CRRT is an effective treatment for patients with MALA with hemodynamic instability. Initial therapy for MALA is resuscitation and supportive care. There is no specific treatment for MALA and sodium bicarbonate alone is generally not sufficient to correct acidosis. Metformin has a low molecular weight and its protein binding rate is limited; therefore, it freely diffuses through hemodialyzers and hemofilters [4]. Intermittent hemodialysis has been suggested as a treatment for drug removal and the correction of severe acidemia. CRRT for the treatment of MALA has been documented in some case reports [13]. The clearance of metformin may exceed 200 mL/minute with intermittent dialysis and up to 50 mL/minute with CRRT [5, 14]. The clearance of drugs by CRRT may be less effective than that for intermittent hemodialysis, but needs to be considered for patients who are hemodynamically unstable. In our patient, intermittent dialysis was difficult because she was hemodynamically unstable to receive high doses of vasopressors. After CRRT was initiated, her lactate level and pH value improved and she subsequently recovered from shock. CRRT is an effective treatment for MALA if intermittent hemodialysis cannot be performed due to hemodynamic instability.

The most common symptoms of MALA are those related to the gastrointestinal tract (including nausea, vomiting, and diarrhea) followed by an altered mental status, shortness of breath, hypothermia, and hypotension [1]. Metformin toxicity may mimic sepsis with gastrointestinal symptoms. MALA is characterized by a blood lactate concentration greater than 5 mmol/L and arterial pH less than 7.35 in association with metformin exposure. Metformin clearance decreases with reductions in the glomerular filtration rate because metformin has limited metabolism and is mostly eliminated unchanged by the kidney [15]. In the present case, her serum metformin concentration was elevated because of prerenal acute renal failure induced by repeated diarrhea and vomiting. It was not clear whether repeated vomiting and diarrhea were due to enteritis or MALA; however, the present case demonstrated that MALA may occur even if patients take an appropriate amount of metformin. In emergency settings, difficulties may be associated with obtaining a detailed medical history; therefore, it is important to suspect MALA in patients with metabolic acidosis and elevated lactate levels in any setting. In the present case, we obtained a detailed medical history, which included a history of type 2 diabetes mellitus, treatment with metformin, and a history of preceding gastrointestinal symptoms; therefore, we immediately suspected MALA.

We described one of the highest metformin concentrations in MALA successfully treated with CRRT. Since MALA is difficult to differentiate from sepsis, particularly in severe cases, serum metformin concentrations may be useful for reaching a diagnosis. MALA needs to be considered a differential diagnosis in patients with type 2 diabetes mellitus receiving metformin even without overdose who have severe lactic acidosis. Although intermittent hemodialysis is the preferred approach, CRRT is an effective alternative for hemodynamically unstable patients.

## Data Availability

Not applicable.

## Abbreviations

CRRT: Continuous renal replacement therapy
MALA: Metformin-associated lactic acidosis
SLED: Sustained low-efficacy dialysis
